# Overexpression of THI4 and HAP4 Improves Glucose Metabolism and Ethanol Production in *Saccharomyces cerevisiae*

**DOI:** 10.3389/fmicb.2018.01444

**Published:** 2018-06-27

**Authors:** Xinchi Shi, Yanan Zou, Yong Chen, Hanjie Ying

**Affiliations:** ^1^National Engineering Research Center for Biotechnology, College of Biotechnology and Pharmaceutical Engineering, Nanjing Tech University, Nanjing, China; ^2^School of Life Sciences, Nantong University, Nantong, China; ^3^State Key Laboratory of Materials–Oriented Chemical Engineering, Nanjing Tech University, Nanjing, China; ^4^Jiangsu National Synergetic Innovation Center for Advanced Materials, Nanjing, China

**Keywords:** *Saccharomyces cerevisiae*, THI4, HAP4, NAD(H), osmotolerance

## Abstract

Redox homeostasis is essential to the maintenance of cell metabolism. Changes in the redox state cause global metabolic and transcriptional changes. Our previous study indicated that the overexpression of NADH oxidase in *Saccharomyces cerevisiae* led to increased glucose consumption and ethanol production. Gene expression related to thiamine synthesis and osmotolerance as well as *HAP4* expression was increased in response to redox change caused by the overexpression of NADH oxidase. To identify detailed relationships among cofactor levels, thiamine synthesis, expression of HAP4, and osmotolerance, and to determine whether these changes are interdependent, THI4 and HAP4 were overexpressed in *S. cerevisiae* BY4741. The glucose consumption rate of THI4-overexpressing strain (thi4-OE) was the highest, followed by HAP4-overexpressing strain (hap4-OE) > NADH oxidase-overexpressing strain (nox-OE) > control strain (con), while strain hap4-OE showed the highest concentration of ethanol after 26 h of fermentation. Reduced glycerol production and increased osmotolerance were observed in thi4-OE and hap4-OE, as well as in nox-OE. HAP4 globally regulated thiamine synthesis, biomass synthesis, respiration, and osmotolerance of cells, which conferred the recombinant strain hap4-OE with faster glucose metabolism and enhanced stress resistance. Moreover, overexpression of HAP4 might extend the life span of cells under caloric restriction by lowering the NADH level. Although overexpression of THI4 and HAP4 induced various similar changes at both the metabolic and the transcriptional level, the regulatory effect of THI4 was more limited than that of HAP4, and was restricted to the growth phase of cells. Our findings are expected to benefit the bio-ethanol industry.

## Introduction

Redox homeostasis plays a crucial role in the maintenance of cell metabolism. The intracellular redox potential is primarily determined by the levels of cofactors, including the NADH/NAD^+^ and NADPH/NADP^+^ ratios (Vemuri et al., 2007). Additionally, thiamine (vitamin B1), the active form of which is ThDP, is an important metabolic cofactor that can alleviate redox stress in *Saccharomyces cerevisiae* (Alff-Tuomala et al., 2016). 5-Hydroxyethyl-4-methylthiazole (HET), formed from ADT by thiazole synthase (THI4) (Faou and Tropschug, 2004), is a precursor of thiamine, and NAD^+^ is the precursor of ADT (Jurgenson et al., 2006; Chatterjee et al., 2007). HMP is another precursor of thiamine. The THI5 family (THI5, THI11, THI12, and THI13) catalyzes the reaction from pyridoxine (VB6) to HMP (Wightman and Meacock, 2003). Previous studies on THI4 have mainly focused on the intermediate products generated and the enzymes involved in the synthetic process (Jurgenson et al., 2006; Chatterjee et al., 2007); however, the influence of THI4 on metabolism remains unclear. Wightman and Meacock (2003) found that as a response to increased ThDP demand, the expression of the *THI5* gene family is increased, which increases the flux to the Embden–Meyerhof pathway. In our previous study (Shi et al., 2016b), we found that along with increased glucose-consumption and cell-growth rates in an NADH oxidase-overexpressing *S. cerevisiae* strain (nox-OE), the expression levels of *THI4* and *THI5* were significantly increased in the mid-exponential phase. Moreover, the osmotolerance of the cells was increased, likely because of the enhanced thiamine synthesis. NADH oxidase catalyzes the conversion of NADH to NAD^+^, thus increasing the regeneration of NAD^+^, which in turn provides more precursor for the reaction catalyzed by THI4. We proposed a relationship of the accelerated fermentation and increased osmotolerance with increased *THI* gene expression as a response to the alleviated redox stress by NADH oxidase overexpression (Shi et al., 2016b), but the detailed mechanism and interaction between the pathways involved were not clarified.

The global transcriptional activator HAP4 is required for the induction of respiratory activity in the mitochondria and can induce physiological changes, such as those involved in the diauxic shift (Lascaris et al., 2002). Overexpression of HAP4 in *S. cerevisiae* resulted in increased mitochondrial biogenesis and respiratory activity in glucose growth conditions (Lascaris et al., 2002). In our previous study, the expression of HAP4 was significantly increased in the late-exponential phase, and the expression level of the yeast apoptosis-inducing factor NUC1 was decreased throughout fermentation progress in nox-OE as compared to that in the control strain (con) (Shi et al., 2016b). Moreover, in the condition of enhanced mitochondrial respiration owing to HAP4 overexpression, the downregulation of NUC1 inhibited apoptosis (Carmona-Gutierrez et al., 2010), which may be related to the increased osmotolerance.

THI4 and THI5 catalyze specific reactions in the metabolic network of *S. cerevisiae*. While THI4 is associated with NAD^+^ (Jurgenson et al., 2006; Chatterjee et al., 2007), THI5 is not (Wightman and Meacock, 2003). NADH/NAD^+^ is involved in a multitude of reactions spanning a wide range of cellular functions in *S. cerevisiae* (Doerks et al., 2002), and any change in the NADH/NAD^+^ ratio leads to widespread metabolic changes (Nielsen, 2003). It can be assumed that a change in THI4 expression can widely affect metabolism; however, whether overexpression of THI4 causes a global change in the HAP4 transcription level through the cofactors NAD^+^ and ThDP remains to be confirmed. Changes in metabolism and transcription caused by the overexpression of these genes also need to be compared with those caused by the overexpression of NADH oxidase.

To further study the relationships between cofactor levels, thiamine synthesis, HAP4 and THI4 expression, osmotolerance, and the regulatory mechanisms of HAP4 and THI4 in *S. cerevisiae*, THI4, THI5, and HAP4 were overexpressed in *S. cerevisiae*.

## Materials and Methods

### Construction of the Yeast Strains

The primers used in this study are listed in **Table 1**. The *THI4* gene (GenBank Accession No. NC001139) from *S. cerevisiae* BY4741 was amplified with primers having an *Eco*RV site. The *HAP4* gene (GenBank Accession No. NC001143) from *S. cerevisiae* BY4741 was amplified with primers having a *Eco*RV site.

**Table 1:** Primers used in this study.

textbfName	Sequence (5′→3′)	Template
THI4-F	*GCTAGCTGAGCTCGA*GATATCATGTCTGCTACCTCTAC	Genomic DNA of *S. cerevisiae* BY4741
THI4-R	*GTGCCTGACTACGCAT*GATATCCTAAGCAGCAAAGTGTTTC	
TEF1-F	*GCTTGTGGGCCCTA*GGATCCGATCCCCCACACACC	Plasmid DNA of pGAPZα
TEF1-R	*GATCTTGTCTGTAGACAT*CTTAGATTAGATTGCTATG	
THI5-F	*CATAGCAATCTAATCTAAG*ATGTCTACAGACAAGATC	Genomic DNA of *S. cerevisiae* BY4741
THI5-R	*CATAACTAATTACATGA*TTAAGCTGGAAGAGCC	
CYC-F	*GGCTCTTCCAGCTTAA*TCATGTAATTAGTTATGTC	Plasmid DNA of pGAPZα
CYC-R	*CATACAGGAATTCACCAT*GGATCCGCAAATTAAAGCCTTCG	
HAP4-F	*GCTAGCTGAGCTCGA*GATATCATGACCGCAAAGACTTTTC	Genomic DNA of *S. cerevisiae* BY4741
HAP4-R	*GTGCCTGACTACGCAT*GATATCTCAAAATACTTGTACC	

*The restriction enzyme site is underlined and the homologous arms are indicated in italics in the primer sequences.*

The primers for TEF1-F and CYC-R had a *Bam*HI site. Gel-purified PCR products of the TEF1 promoter, *THI5* gene (GenBank Accession No. NC001138, encoding 4-amino-5-hydroxymethyl-2-methylpyrimidine phosphate synthase), and CYC terminator were concatenated by overlap PCR. PCR products were gel-purified and inserted into pYX212 by using the ClonExpress One Step Cloning Kit (Vazyme Biotech, Nanjing, China). The plasmids were transformed into the host strain, BY4741, using G418 (400 μg/mL) to select stably transfected clones. The strains and plasmids used in this study are listed in **Table 2**. As a control, con, the host strain transfected with empty plasmid, was used.

**Table 2:** Plasmids and strains used in this study.

Plasmid/strain	Genotype	Source
**Plasmid**		
pYX212	2 μ, TPI promoter, AmpR, KanR	A gift from Prof. Yingjin Yuan (Tianjin University, Tianjin, China)
pYX212-THI4	pYX212 with *THI4* from *S. cerevisiae* BY4741	This study
pYX212-THI4-THI5	pYX212 with *THI4* and *THI5* (controlled by TEF1 promoter) from *S. cerevisiae* BY4741	This study
pYX212-HAP4	pYX212 with *HAP4* from *S. cerevisiae* BY4741	This study
**Strain**		
BY4741	*MATa;ura3;his3;leu2;met15*	A gift from Prof. Yingjin Yuan (Tianjin University, Tianjin, China)
con	BY4741/pYX212	Shi et al., 2016a
nox-OE	BY4741/pYX212-NOX	Shi et al., 2016a
thi4-OE	BY4741/pYX212-THI4	This study
thi4-thi5-OE	BY4741/pYX212-THI4-THI5	This study
hap4-OE	BY4741/pYX212-HAP4	This study

### Media and Growth Conditions

The strains were maintained on conventional yeast extract peptone dextrose agar plates as described previously (Ito et al., 1983). Seed cultures for cultivation were grown at 30°C in 500-mL Erlenmeyer flasks containing 100 mL complex medium A (initial pH 5.2) containing glucose (20 g/L), tryptone (10 g/L; oxoid), yeast extract (5 g/L; oxoid), and NaCl (9 g/L) (Barber et al., 2002) on a rotary shaker at 200 rpm. Aerobic fermentations were performed at 32°C in 500-mL Erlenmeyer flasks containing 100 mL complex medium A with 100 g/L glucose (initial pH 5.2) on a rotary shaker at 200 rpm after the addition of cells obtained by centrifugation of 10 mL of OD-standardized seed cultures. G418 (400 μg/mL) was used as a selection agent.

### Metabolite Analyses

Cell density was measured using a BioMate 3 spectrophotometer (Thermo Scientific, Waltham, MA, United States) at 600 nm. Five milliliters of culture was centrifuged at 4,000 ×*g* for 10 min. The supernatants were used to determine the concentrations of glucose, ethanol, glycerol, and acetic acid.

Glucose and glycerol concentrations were measured by high-performance liquid chromatography (HPLC; Agilent 1100 series; Hewlett–Packard, Palo Alto, CA, United States) with a refractive index detector, using a Benson BP-100 Pb^++^ column (300 × 7.8 mm; Benson Polymeric, Sparks, NV, United States). Ultrapure water was used as the mobile phase at a flow rate of 0.4 mL/min at 80°C. The acetic acid concentration was measured by HPLC (Agilent 1100 series; Hewlett–Packard) with an ultraviolet-visible light absorbance detector, using an Aminex HPX-87H ion exchange column (Bio-Rad, Hercules, CA, United States) at 65°C, using 5 mM H_2_SO_4_ as mobile phase at a flow rate of 0.6 mL/min (Hou et al., 2009).

The ethanol concentration was analyzed by gas chromatography using an Agilent HP-INNOWAX column (60 m × 250 μm × 0.5 μm) with a flame ionization detector, as described previously (Chen et al., 2014).

To measure thiamine content, a small volume (8 mL) of culture was centrifuged and the supernatant was discarded. The cell pellet was washed with deionized water twice and 0.1 mol/L HCl was added. The mixture was shaken and then autoclaved at 121°C of 30 min. Then, the sample was centrifuged again, and the supernatant was filtered through a 0.22-μm filter before use. Thiamine content was measured by HPLC (Agilent 1100 series; Hewlett–Packard) with an ultraviolet-visible light absorbance detector at a wavelength of 238 nm, using a HP-C18 column (4.6 × 250 mm; Sepax Technologies, Newark, NJ, United States). The mobile phase was water:methanol = 7:3 (v/v) with a flow rate of 0.8 mL/min at 35°C. The protocol was carried out in the dark.

### Measurement of Growth Ability

To test the osmotolerance of recombinant strains, cells were cultured on plates with different concentrations of NaCl (9, 18, 36, and 54 g/L) (Xu et al., 2010; Shi et al., 2016b). We previously showed that growth on 0 g/L and 9 g/L NaCl plates is similar (Shi et al., 2016b). Because 9 g/L NaCl mimics the normal physiological state, we used this concentration as a control in this study. Yeast-cell suspensions were OD-standardized before sample application (2 μL).

### Osmolarity Determination

Osmolarity was measured using an automatic cryoscopic osmometer (OSMOMAT 030; GONOTEC GmbH, Berlin, Germany) (Shi et al., 2016b).

### Quantification of Intracellular NAD(P)H/NAD(P)

Intracellular concentrations of NAD(P)H were determined by the enzyme cycling method of Liu et al. (2013) with modifications. In brief, 1-mL cell samples were collected and the cells were dissolved in 0.5 mL 0.1 M NaOH [to assay NAD(P)H] and 0.5 mL 0.1 M HCl [to assay NAD(P)], respectively. The lysates were heated at 50°C for 10 min, cooled to 0°C, and centrifuged at 10,000 ×*g* for 10 min. The supernatants were used for measurements. One hundred microliters of Tris-HCl (1 M, pH 7.8), 100 μL of 4.2 mM MTT, 150 μL of 16.6 mM PES, and 100 μL of ethanol for the determination of NAD(H) or 100 μL of 60 mM glucose 6-phosphate for the determination of NADP(H), were sequentially added and incubated at 37°C in the dark for 5 min. ddH_2_O and an appropriate amount of supernatant (75 μL in total) were added to 96-well plates. The plates were transferred into a Multi-Mode Detection Platform (SpectraMax Paradigm; Molecular Devices, San Jose, CA, United States) and preheated at 37°C for 5 min. Ten microliters of alcohol dehydrogenase [1.5 units/μL, for NAD(H)] or glucose 6-phosphate dehydrogenase [70 units/mL, for NADP(H)] were added to the mixture, and 46 μL of the mixture was added to the 96-well plates to start the reaction. The absorbance at 570 nm was determined. NADH was measured over 10 min with 2-min intervals, and NADPH, NADP, and NAD were measured over 30 min with 5-min intervals (Shi et al., 2016a,b).

### Quantitative Reverse Transcription (qRT)-PCR Analysis

Cells of con, nox-OE, THI4-overexpressing strain (thi4-OE), and HAP4-overexpressing strain (hap4-OE) were collected after 22 h of fermentation by centrifugation (5,000 × *g*, 6 min, 4°C) and were washed twice in PBS (8 g/L NaCl, 0.2 g/L KCl, 1.44 g/L Na_2_HPO_4_, 0.24 g/L KH_2_PO_4_, pH 7.4). The cell pellets were immediately frozen in liquid nitrogen and stored at -80°C. Three samples were pooled and homogenized, and total RNA was extracted to prepare staged samples (Li et al., 2015). Reverse transcription was performed using the AMV First Strand cDNA Synthesis Kit (Sangon Biotech, Shanghai, China) according to the manufacturer’s instructions. Primer Express software was used for primer design. The target genes and primers used in the analysis are listed in **Table 3**. qRT-PCR assays were performed with the SYBR Premix Ex Taq II (Takara Biomedical Technology, Beijing, China) on a StepOnePlus Real-Time PCR System, according to the manufacturer’s instructions. The reaction mixture contained 10 μL SYBR Premix Ex Taq (2×), 0.8 μL forward primer (10 μM), 0.8 μL reverse primer (10 μM), 1 μL cDNA, and ddH_2_O (up to 20 μL). The thermal cycles were: 95°C for 30 s, followed by 40 cycles of 95°C for 5 s and 60°C for 20 s. Fluorescence intensity was detected at 72°C in each cycle. A melting curve was generated by denaturation at 95°C for 15 s, followed by an increase from 65°C to 95°C using the standard ramp rate, with continuous measurement of the fluorescence intensity. Three technical replicates were included for each sample. Gene transcript levels were determined by the 2^-ΔΔC_t_^ method, using *ACT1* (Yuan and Ching, 2015) as a reference gene for normalizing the gene expression levels. To verify qRT-PCR data, standard deviations were calculated using Microsoft Excel (Microsoft Corporation, Redmond, WA, United States) and were used to evaluate the repeatability and the reliability of the data.

**Table 3:** Target genes and primers used for qPCR.

Gene ID	Gene name	Primer sequences (5′→3′)
YFL039C	*ACT1*	F: TGGATTCCGGTGATGGTGTT
	(conference gene)	R: TGGCGTGAGGTAGAGAGAAACC
YDR343C	*HXT6*	F: CGCTGCTATTGCAGAGCAAAC
		R: CGAGTGGGAGGCTGAGTCA
YGR144W	*THI4*	F: TTTGCCGTTTCTGACGTGATT
		R: GCGGCGGATAAACCTGAA
YFL058W	*THI5*	F: GGTTACTTCAAGGAGCAAGGTCTAGA
		R: CAGTGACATCGGAAGGATTGG
YKL109W	*HAP4*	F: TGTACCGATCGCCCCAAATA
		R: TGCCATCGTTTTCGAATTCC
YJL208C	*NUC1*	F: TCGATCCTTCCGGGTTCTT
		R: CGCGGTTCTGCAGATCATG
YDL022W	*GPD1*	F: TCAATTTTTGCCCCGTATCTG
		R: GATAGCTCTGACGTGTGAATCAACA
YHL032C	*GUT1*	F: GCCCCAGCTCGTGAAACA
		R: GGGCTTTCCGCTGGTTTT
YOL086C	*ADH1*	F: GAAGGTGCCGGTGTCGTT
		R: ACCGATCTTCCAGCCCTTAAC
YPL061W	*ALD6*	F: GACAAAGTCAACGGTAGAACAATCA
		R: GGCTCTAAGGTGGTGAAGTTCATG
YLR113W	*HOG1*	F: GGGCATTTGGGTTGGTTTG
		R: TTAATGGCAACTGGCTGAGATG
YML120C	*NDI1*	F: GAGGCCGCTGGTGAACTACA
		R: GCCAATGCAGGGAGAAACTTT
YPR002W	*PDH1*	F: CTTACCGTCATGCCCCAAAT
		R: AGCCCTCGAAACTGCATCTC
YER148W	*SPT15*	F: ACATGCCCGTAATGCAGAATATAA
		R: GGCTCTCTAATACGCATGATGACA
YKL062W	*MSN4*	F: CGGCATTCGACAATAACGTAGA
		R: GATCCTGAGCCGGAGATGAC
YDL042C	*SIR2*	F: GGCAGTGTCAGCAGCTTCAG
		R: GGGCGTCTCTGGTTTCAAAA

### Statistical Analysis

Each measurement was conducted in triplicate. Results are reported as the mean and error of the mean from three replicates. Means of osmolarity and thiamine content between groups were compared using Dunnett’s *t*-test, with the significance threshold set at *P* < 0.05.

## Results and Discussion

### THI4 Overexpression Improves Glucose Fermentation

**FIGURE 1:**
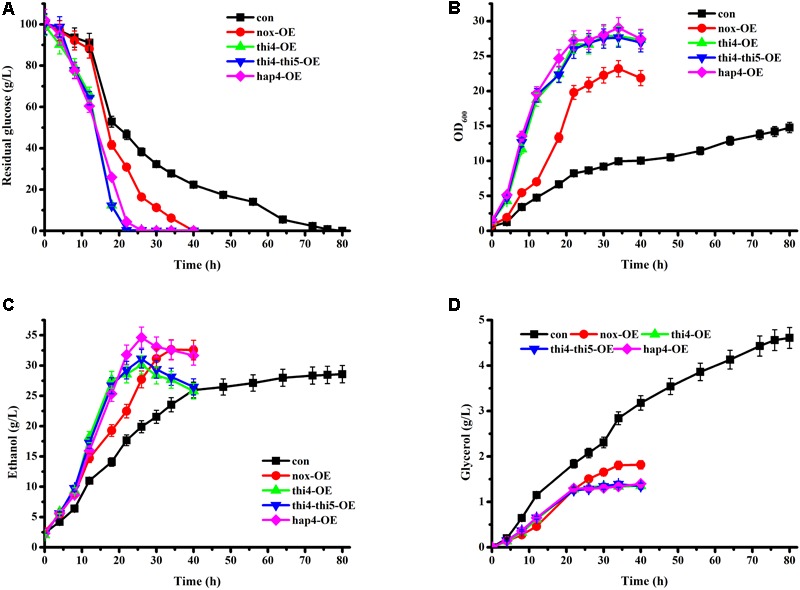
Time course analyses of parameters of batch fermentation by recombinant strains. **(A)** Residual glucose; **(B)** OD_600_; **(C)** ethanol; **(D)** glycerol.

Although THI5 was not the main focus in this study, we overexpressed THI5 in THI4-overexpressing strain (thi4-thi5-OE) to detect any additional effect of this protein on glucose metabolism. Batch cultures of con, nox-OE, thi4-OE, and thi4-thi5-OE strains grown under aerobic conditions were compared (**Figure 1**). The con strain consumed the glucose within 80 h. In accordance with our previous study, the glucose consumption and cell growth rates of nox-OE were higher than those of con (Shi et al., 2016a,b). The glucose consumption and cell growth rates of thi4-OE and thi4-thi5-OE were higher than those of nox-OE and con. Fermentation profiles of thi4-OE and thi4-thi5-OE were similar, which indicates that overexpression of THI4 might affect THI5 expression and further stimulation of the specific reaction catalyzed by THI5 does not enhance thiamine synthesis. However, the exact function of THI5 requires further study. Glucose was exhausted at 22 h by thi4-OE, while 46.56 ± 2.33 g/L and 30.87 ± 1.54 g/L residual glucose remained in the con and nox-OE cultures, respectively, at this time point. Additionally, after 26 h of fermentation, the concentration of ethanol produced by thi4-OE peaked at 30.12 ± 1.51 g/L, which was 51.36% higher than that in con (19.90 ± 0.99 g/L) and 8.66% higher than that in nox-OE (27.72 ± 1.38 g/L) culture at this time point. Consistent with our previous study, glycerol production by nox-OE was lower than that by con (Shi et al., 2016a,b), and thi4-OE produced slightly less glycerol than did nox-OE. Although THI4 uses NAD^+^ as a precursor, NADH is not produced in this reaction, and the increased NAD^+^ demand might stimulate the oxidation of NADH to NAD^+^, which might alleviate NADH accumulation because of rapid glycolysis (Vemuri et al., 2007), supported by the inactivation of the glycerol synthesis pathway. Different from our previous studies (Shi et al., 2016a,b), we centrifuged the seed culture and discarded the supernatant before adding the cells to the fermentation medium, as we had observed that abundant glycerol was produced during seed culture growth. Adding cells alone eliminated this effect; the production of glycerol by con during fermentation was indeed decreased.

### HAP4 Overexpression Improves Glucose Fermentation

Batch cultures of hap4-OE and the other strains grown under aerobic conditions were compared (**Figure 1**). The glucose consumption and cell growth rates of hap4-OE were higher than those of nox-OE and con, but slightly lower than those of thi4-OE and thi4-thi5-OE. After 26 h of fermentation, the concentration of ethanol produced by hap4-OE peaked at 34.63 ± 1.73 g/L, which was the highest among the five strains and 14.97% higher than that of thi4-OE. Although the concentrations of ethanol after overexpression of NADH oxidase, THI4, or HAP4 were increased, the yields (0.39, 0.30, 0.34 g/g glucose, respectively) were decreased as compared to con (0.42 g/g glucose), which was in line with the results of Blom et al. (2000). Overexpression of HAP4 increases the respiration of *S. cerevisiae* cells (Lascaris et al., 2002). In the oxidation of NADH, a large amount of energy is released. Overexpression of NADH oxidase stimulates the oxidation of NADH to NAD^+^, thereby producing H_2_O rather than other metabolites. Complexes I and IV in the electron transport chain oxidize NADH to NAD^+^, while H_2_O is produced at complex IV. Much of the energy released via the oxidation of NADH was used to produce H_2_O, but not to the benefit of ethanol production in nox-OE and hap4-OE, which might be the main reason for the decreased ethanol yield. In line with the findings of Blom et al. (2000), glycerol production by hap4-OE was lower than that by con, and similar to that by thi4-OE. This change in product pattern was attributed to an increased ability to oxidize redox equivalents via respiration (Blom et al., 2000), which could also alleviate the accumulation of NADH.

### NADH/NAD^+^ and NADPH/NADP^+^ Levels in Recombinant Strains

**FIGURE 2:**
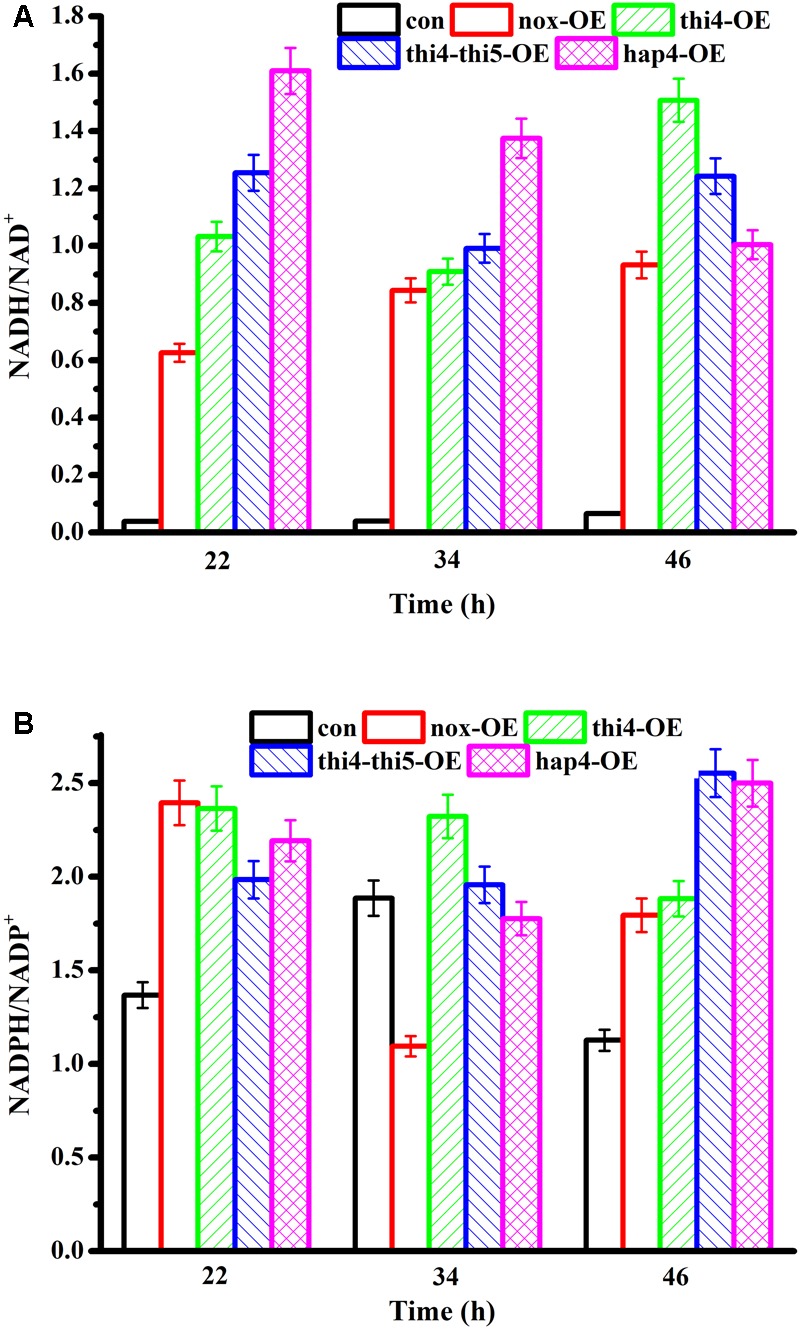
**(A)** NADH/NAD^+^ and **(B)** NADPH/NADP^+^ ratios of recombinant strains. In the batch fermentation, the concentrations of the intracellular cofactors of con, nox-OE, thi4-OE, and hap4-OE were measured at 22, 34, and 46 h.

The levels of redox cofactors in the recombinant cells were determined after 22, 34, and 46 h of fermentation (**Figure 2**). At 22 h and 34 h, the NADH/NAD^+^ ratio of hap4-OE was the highest, followed by those of thi4-thi5-OE > thi4-OE > nox-OE > con. We previously found that the NADH/NAD^+^ ratio is not determined by a single reaction (Shi et al., 2016a,b), and in agreement with our previous findings, the increased glucose consumption rate was attributed to the increased NADH/NAD^+^ ratio, whether the overexpressed proteins consumed NAD(H) directly or indirectly. However, although the thi4-thi5-OE and thi4-OE strains showed a higher glucose consumption rate than hap4-OE, their NADH/NAD^+^ ratios were lower than that of hap4-OE, indicating that the glycolytic rate was also one of the factors that affected the NADH/NAD^+^ ratio. Glucose was exhausted in thi4-thi5-OE, hap4-OE, and nox-OE cultures at 22, 26, and 40 h, respectively. After the glucose was exhausted, the NADH/NAD^+^ ratio of hap4-OE was decreased, while the NADH/NAD^+^ ratios of thi4-OE, thi4-thi5-OE, and nox-OE were higher at 46 h than at 34 h. Overexpression of HAP4 increased cell respiration, which led to a decrease in NADH levels (Lin et al., 2004). There was no obvious pattern in the changes in NADPH/NADP^+^ of the five strains.

### Relationship of Overexpression of THI4 and HAP4 With Thiamine Synthesis

Our previous study revealed that the overexpression of NADH oxidase in *S. cerevisiae* led to an increase in the expression of genes involved in thiamine synthesis in the mid-exponential phase and of HAP4 in the late-exponential phase (Shi et al., 2016b). To further study the relationship between the redox condition and expression of HAP4 and thiamine synthesis, the thiamine contents of the recombinant strains were measured after 22 h of fermentation (**Table 4**). The thiamine contents of hap4-OE and nox-OE were higher than that of thi4-OE, indicating that overexpression of HAP4 and NADH oxidase on intracellular thiamine accumulation was more effective than THI4 overexpression, or that thiamine might exist in the form of ThDP in thi4-OE, in which the consumption of thiamine/ThDP might be higher than in other strains. The level of thiamine in con was undetectable, which confirmed that thiamine was involved in the regulation of the redox changes caused by overexpression of the specific genes (Alff-Tuomala et al., 2016).

**Table 4:** Thiamine content and osmotic pressure of the fermentation fluid after 22 h of fermentation.

	con	nox-OE	thi4-OE	hap4-OE
Thiamine (nmol/L/OD_600_)	0.00 ± 0.00	92.23 ± 0.065^∗^	74.06 ± 0.18^∗^	132.58 ± 0.57^∗^
Osmolarity (mOsmol/kg)	1348.00 ± 1.00	1466.00 ± 1.00^∗^	1504.00 ± 1.00^∗^	1542.00 ± 2.00^∗^

*Each value is a mean ± standard deviation of three replicates. ^∗^*P* < 0.0001, Dunnett’s *t*-test.*

### Relationship of Overexpression of THI4 and HAP4 With Osmotolerance

We previously found that the overexpression of NADH oxidase increased the osmotolerance of *S. cerevisiae* cells (Shi et al., 2016b). However, the relationship between thiamine, HAP4, and osmotolerance was not clarified. In this study, osmolarity of cultures was measured after 22 h of fermentation (**Table 4**). The osmolarity of hap4-OE was the highest, followed by thi4-OE > nox-OE > con, which was in line with the concentrations of ethanol (**Figure 1C**), as expected. The higher osmolarity of the thi4-OE and hap4-OE strains did not weaken their metabolism, as indicated by the higher glucose consumption and cell growth rates.

**FIGURE 3:**
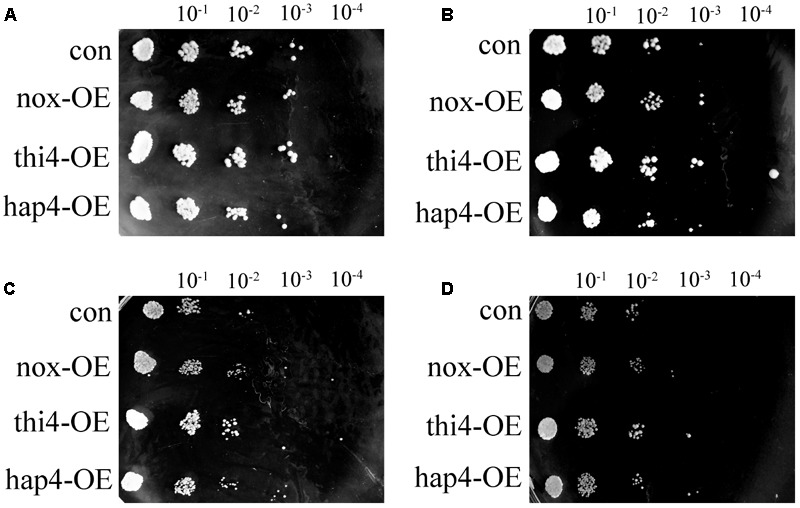
Cell growth of recombinant strains in yeast extract peptone dextrose plates with different concentrations of NaCl. **(A)** 9 g/L; **(B)** 18 g/L; **(C)** 36 g/L; **(D)** 54 g/L.

To further study the survival ability of recombinant strains under hyperosmotic stress, qualitative sensitivity at different concentrations of NaCl was measured as colony-forming units at different dilution levels (**Figure 3**) (Xu et al., 2010). After 48 h, the four strains developed similar colonies at different dilutions of 9 g/L of NaCl, which represents the normal physiological condition (**Figure 3A**). With increasing concentration of NaCl, differences in survival ability became apparent. The engineered nox-OE, thi4-OE, and hap4-OE strains had a 100-fold higher survival rate than con at 36 g/L of NaCl (**Figure 3C**). At 54 g/L of NaCl (**Figure 3D**), hap4-OE showed a 100-fold higher survival rate than con, and a 10-fold higher survival rate than nox-OE and thi4-OE. These results suggested that overexpression of NADH oxidase, THI4, and HAP4 in *S. cerevisiae* confers protection under hyperosmotic conditions, and the increased osmotolerance of nox-OE cells was indeed related with the increased expression of thiamine synthesis genes and HAP4 observed in our previous study (Shi et al., 2016b).

### Key Gene Transcription in Response to Overexpression of THI4 and HAP4

The transcription levels of various key genes in the recombinant strains were verified by qRT-PCR after 22 h of fermentation (**Table 5**). In line with the results of batch fermentation (**Figure 1**), the expression levels of *GPD1* involved in the glycerol synthetic pathway were decreased, while those of *ADH1* involved in the ethanol production pathway were increased in nox-OE, thi4-OE, and hap4-OE as compared to those in con. In accordance with thi4-OE showing the highest glucose consumption rate (**Figure 1A**), the expression level of the high-affinity hexose transporter *HXT6* was the highest in thi4-OE. The expression level of *THI5* was increased in thi4-OE, which might explain why overexpression of THI5 in this strain had a very limited effect. The expression of *HAP4* was increased in thi4-OE, mainly because the cells underwent a diauxic shift, which was in accordance with the results of our previous study that *HAP4* expression in nox-OE increased when glucose was exhausted (Shi et al., 2016b). The expression levels of *THI4* and *THI5* involved in thiamine synthesis in the hap4-OE and thi4-OE strains were higher than those in nox-OE and con, and hap4-OE showed the highest expression levels of these two genes among the four strains.

**Table 5:** Relative expression of key genes in the recombinant strains.

Genes	Gene expression in recombinant strains relative to con		
	nox-OE	thi4-OE	hap4-OE
*ADH1*	1.07	3.56	4.10
*ALD6*	3.19	3.39	3.66
*GPD1*	0.68	0.58	0.58
*GUT1*	0.94	1.49	0.87
*HAP4*	0.92	4.85	14.49
*HXT6*	1.17	234.48	45.36
*HOG1*	0.58	3.01	2.19
*THI4*	3.80	10.06	35.92
*THI5*	1.48	3.66	20.11
*NUC1*	0.99	1.92	4.12

### Regulatory Mechanism of HAP4

**FIGURE 4:**
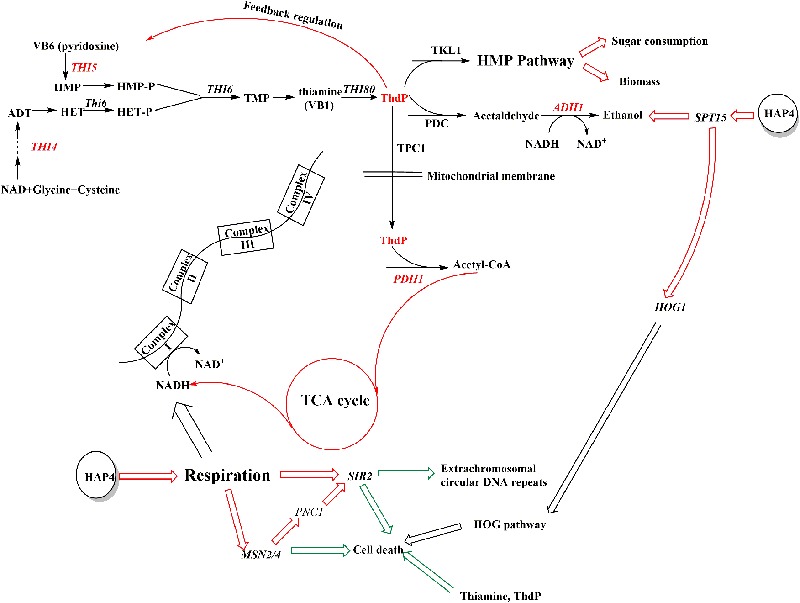
Overexpression of HAP4 in *S. cerevisiae* enhances thiamine synthesis, osmotolerance, and cell viability. The red mark highlights the effect of acceleration, while the green arrow highlights the effect of reduction. Genes indicated in red were upregulated in hap4-OE vs. con.

As most of the changes in thi4-OE and hap4-OE were similar, we chose hap4-OE to study the mechanisms underlying these changes. Complex I of the electron transport chain consumes NADH, which is produced by the tricarboxylic acid cycle (**Figure 4**). Overexpression of HAP4 in *S. cerevisiae* enhances the biosynthesis of mitochondria, and thus, respiration (Lascaris et al., 2002). The increased respiration increases the NADH demand, which in turn boosts the tricarboxylic acid cycle, the initial substrate of which is acetyl-CoA. Respiration was enhanced in hap4-OE, thi4-OE, and nox-OE, albeit to different levels. The carbon flux was altered to respiration rather than ethanol production, which explains the increased glucose consumption rates and ethanol concentrations, and decreased ethanol yields of overexpression strains as compared to con. The conversion of pyruvic acid to acetyl-CoA is catalyzed by pyruvate dehydrogenase (PDH1), which requires ThDP as a cofactor (Hohmann and Meacock, 1998). ThDP can regulate the expression of most genes involved in thiamine synthesis via a feedback mechanism (Wightman and Meacock, 2003). The enhanced respiration in hap4-OE increased the demand for ThDP, which in turn increased the expression of *THI4* and *THI5* (**Table 5**). Increased *THI5* expression promotes the expression of hexose transport proteins (such as *HXT6*), which leads to improved absorption and metabolism of carbon (Wightman and Meacock, 2003).

In addition to PDH1, pyruvate decarboxylase (PDC) also requires ThDP to convert pyruvic acid to acetaldehyde (Hohmann and Meacock, 1998). The increased amount of ThDP might have promoted this reaction, which might have benefited ethanol production. In addition, ThDP is a cofactor for transketolase (TKL1) in the pentose phosphate pathway (Hua et al., 1999). This pathway is the main way for NADPH production, which has great significance to maintaining cell growth and carbon metabolism (Hua et al., 1999). Overexpression of HAP4 promoted ThDP synthesis, thus promoting sugar consumption and biomass synthesis (**Figure 4**).

Overexpression of HAP4 enhances respiration and reduces NADH accumulation, thus alleviating the inhibitory effect of NADH on the histone deacetylase, SIR2 (Piper et al., 2006). Moreover, the enhanced respiration promotes the expression of the transcription factor MSN2/4, which induces the expression of *PNC1* to activate SIR2 (Vendrell et al., 2011). SIR2 activation leads to reduced extrachromosomal circular DNA repeats, which extend the life span of cells, and this function mainly works through a decrease in the level of NADH under the condition of calorie restriction (Lin et al., 2004). As can be seen from the results for NADH/NAD^+^ ratios in **Figure 2**, only hap4-OE tended to have a decreased NADH/NAD^+^ ratio at the three time points measured, indicating that this strain can release energy through reducing the level of NADH, which might help maintaining cellular activity when facing exhaustion of the fermentable carbon source. However, the regulation of HAP4 in *S. cerevisiae* BY4741 under strict caloric restriction could not be determined in this study and requires further research.

SIR2 activation protects yeast cells from cell death. THI4 has a dual function of catalyzing thiazole synthesis and protecting mitochondrial DNA. In the process of thiazole synthesis, the intermediate produced by THI4 can scavenge DNA-damaging free radicals, like glutathione (Hohmann and Meacock, 1998). Increased MSN2/4 expression increases stress resistance and cell survivability in yeast (Piper et al., 2006). Thus, the increased expression of SIR2, THI4, and MSN2/4 might be related to the increased osmotolerance of hap4-OE. This might explain why although *HOG1* expression was higher in hap4-OE than in con (**Table 5**), the osmotolerance experiments (**Figure 3**) showed that HAP4 overexpression improved osmotolerance, which proves that the HOG pathway, a MAPK pathway that responds to hyperosmosis or oxidative stress and plays an important role in the response to hyperosmosis in yeast cells (O’Rourke et al., 2002), was not activated. Moreover, the increased expression of *HOG1* might be the result of the increased expression of *SPT15*, which encodes a TATA-binding protein, and might be responsible for the increased ethanol tolerance of the strain (Kim et al., 2013).

qRT-PCR was performed to analyze the expression of key genes in hap4-OE in comparison with con. The relative expression of *NDI1, PDH1, SPT15, MSN4*, and *SIR2* in hap4-OE was 1.89, 4.84, 3.09, 3.57, and 1.82, respectively. These data support the theory we put forward above. Detailed qRT-PCR data and calculations are shown in Supplementary File 1.

### Comparison of the Regulation by THI4 and HAP4

HAP4 regulates global metabolism and promotes mitochondrial biosynthesis, cell respiration, thiamine synthesis, biomass synthesis, ethanol tolerance, and cell survivability. Although the thi4-OE strain showed a higher glucose consumption rate than hap4-OE, and overexpression of THI4 improved the osmotolerance of cells, the ethanol production by THI4 was lower than that by hap4-OE. As in the nox-OE strain, the expression of HAP4 increased until the glucose was exhausted in thi4-OE, which then induced global regulation of respiration, ethanol tolerance, cell survivability, and expression of genes involved in thiamine synthesis (Piper et al., 2006). These effects caused by HAP4 were not observed throughout the fermentation process of thi4-OE, which limits the regulation of metabolism. Data from our previous study also support this view. The expression levels of the *THI5* family and *THI4* were increased in the mid-exponential phase in nox-OE, while the expression of HAP4 did not significantly change in this phase but was significantly increased in the late-exponential phase, when glucose was exhausted (Shi et al., 2016b). Moreover, different mechanisms might underlie the decreased cell death in the thi4-OE, hap4-OE, and nox-OE strains. Our previous study revealed that the decreased cell death in nox-OE might be connected with decreased expression of the cell death-inducing factor NUC1 and deactivation of the HOG pathway. However, the expression levels of *NUC1* in thi4-OE and hap4-OE were increased (**Table 5**), implying that THI4 and HAP4 regulate cell death via another mechanism. However, the detailed relationship between overexpression of THI4/HAP4 and yeast apoptosis could not be determined in this study and requires further research.

## Conclusion

This study unraveled the response of *S. cerevisiae* to overexpression of THI4 and HAP4 at the metabolic and the transcriptional levels. In batch fermentation, we observed reduced glycerol production and increased glucose consumption, ethanol production, and cell growth in the THI4 and HAP4 overexpression strains, showing their potential usability in large-scale ethanol production. Moreover, osmotolerance was increased in these strains. HAP4 was found to globally regulate thiamine synthesis, biomass synthesis, respiration, and osmotolerance, which conferred the recombinant strain with faster metabolism and enhanced stress resistance. Moreover, overexpression of HAP4 extended the life span of cells through a decrease in the NADH level. Although overexpression of THI4 and HAP4 induced various similar changes at both the metabolic and transcriptional levels, the regulatory activity of THI4 was more limited than that of HAP4, and was restricted to the growth phase of cells. Our findings are expected to benefit the bio-ethanol industry. However, the effect of strain background on the regulatory effects of THI4 and HAP4 requires further research.

## Availability of Data and Material

The datasets supporting the conclusions of this article are included within the article and its Supplementary Files.

## Author Contributions

XS participated in the design of the study, constructed the plasmids and strains, participated in the fermentation experiments, drafted the manuscript, and revised the manuscript. YZ participated in the fermentation experiments. YC participated in the design of the study. HY conceived of the study and participated in its design. All authors have read and approved the final manuscript. All the authors agreed to the publication of this manuscript.

## Conflict of Interest Statement

The authors declare that the research was conducted in the absence of any commercial or financial relationships that could be construed as a potential conflict of interest.

## Abbreviations

ADT: adenosine diphospho-5-(β-ethyl)-4-methylthiazole-2-carboxylic acid
con: *S. cerevisiae* BY4741 containing the empty plasmid pYX212
hap4-OE: *S. cerevisiae* BY4741 overexpressing the transcription factor HAP4
HET: 5-hydroxyethyl-4-methylthiazole
HMP: hydroxymethylpyrimidine
nox-OE: *S. cerevisiae* BY4741 overexpressing the water-forming NADH oxidase encoded by *noxE*
ThDP: thiamine pyrophosphate
thi4-OE: *S. cerevisiae* BY4741 overexpressing the thiazole synthase encoded by *THI4*
thi4-thi5-OE: *S. cerevisiae* BY4741 overexpressing the thiazole synthase encoded by *THI4* and the 4-amino-5-hydroxymethyl-2-methylpyrimidine phosphate synthase encoded by *THI5*
